# Gene Technology for Papaya Ringspot Virus Disease Management

**DOI:** 10.1155/2014/768038

**Published:** 2014-03-17

**Authors:** Md. Abul Kalam Azad, Latifah Amin, Nik Marzuki Sidik

**Affiliations:** ^1^Centre for General Studies, Universiti Kebangsaan Malaysia (UKM), 43600 Bangi, Selangor, Malaysia; ^2^Department of Agricultural Extension, Khamarbari, Farmgate, Dhaka 1215, Bangladesh; ^3^Faculty of Science and Technology, Universiti Kebangsaan Malaysia (UKM), 43600 Bangi, Selangor, Malaysia

## Abstract

Papaya (*Carica papaya*) is severely damaged by the papaya ringspot virus (PRSV). This review focuses on the development of PRSV resistant transgenic papaya through gene technology. The genetic diversity of PRSV depends upon geographical distribution and the influence of PRSV disease management on a sequence of PRSV isolates. The concept of pathogen-derived resistance has been employed for the development of transgenic papaya, using a coat protein-mediated, RNA-silencing mechanism and replicase gene-mediated transformation for effective PRSV disease management. The development of PRSV-resistant papaya via post-transcriptional gene silencing is a promising technology for PRSV disease management. PRSV-resistant transgenic papaya is environmentally safe and has no harmful effects on human health. Recent studies have revealed that the success of adoption of transgenic papaya depends upon the application, it being a commercially viable product, bio-safety regulatory issues, trade regulations, and the wider social acceptance of the technology. This review discusses the genome and the genetic diversity of PRSV, host range determinants, molecular diagnosis, disease management strategies, the development of transgenic papaya, environmental issues, issues in the adoption of transgenic papaya, and future directions for research.

## 1. Introduction

Papaya (*Carica papaya* L) belongs to the family Caricaceaeand is one of the most economically important fruit crops in many tropical and subtropical countries. Papaya is a dicotyledonous, polygamous, and diploid species. The geographical origin of papaya is Southern Mexico and Costa Rica [1]. Papaya has been cultivated in the USA, India, Brazil, Mexico, Nigeria, Jamaica, Indonesia, China, Taiwan, Peru, Thailand, and the Philippines [2]. Papaya fruit is known for its high nutritive and medicinal value [3]. Papaya is rich source of vitamin A, B, and C, as well as proteolytic enzymes like papain and chymopapain. It is an excellent source of beta carotene which may prevent cancer, diabetes, and heart disease [4]. Ripe fruits are usually eaten fresh and can be processed into jam, jelly, marmalade, and candy. The “green” or unripe fruits can be used as vegetables. Papaya is also utilized in the pharmaceutical and cosmetics industries [5].

Papaya crops are currently beset by disease problems especially those caused by the papaya ringspot virus (PRSV) [6, 7]. Symptoms of PRSV manifest as a prominent mosaic pattern on the leaf lamina, wet-oily streaks on the petioles and upper part of the trunk, and the distortion of young leaves. PRSV is the most serious threat to papaya production in the world [8]. PRSV has been recognized as a destructive disease in many tropical and subtropical areas including the USA, South America, Africa [9], India [10], Thailand, Taiwan, China and the Philippines [11], Mexico [12], Australia [13], Japan [14], French Polynesia, and the Cook islands [15] resulting in the decline in fruit production. This disease can cause up to 100% losses of crops in some regions [16]. PRSV is transmitted in a nonpersistent way by several species of aphids in a process involving coat protein (CP) and the helper component proteinase (HC-Pro) [17–19].

Papaya and the aphid transmitted virus are cosmopolitan in distribution [16]. PRSV is controlled by different methods such as rouging of infected plants, use of barrier crops, cross protection, and transgenic resistance [20]. PRSV disease management, via vector controlling, is very difficult to conduct whilst cross protection for controlling PRSV disease is not effective worldwide. Resistance against PRSV has not been found in* Carica papaya* [21]. On the other hand, several wild* Carica *species such as* C. cauliflora, C. pubescens,* and* C. quercifolia* are resistant to PRSV, but these are sexually incompatible with* C. papaya* [22]. The most effective method of controlling plant viruses is through enhancing population resistance [23]. Genetic transformation of plants has made it possible to introduce selected genes into plants for controlling plant diseases and pests. The concept of pathogen derived resistance has stimulated research into obtaining virus resistance in papaya through gene technology. Pathogen-derived resistance is mediated either by proteins encoded by transgenes (protein-mediated) or by the transcripts produced from the transgene (RNA-mediated). Recently, research has indicated that pathogen-derived resistance is mediated by an RNA-based posttranscriptional gene-silencing mechanism. Protein-mediated resistance provides moderate protection against a broad range of related viruses whilst RNA-mediated resistance offers high levels of protection to closely related strains of the virus [24]. The RNAi technology has enabled the induction of an immune reaction to PRSV. This technology has been at the forefront of the new era in the development of eco-friendly molecular tools, which can be suppressed by specific genes which are responsible for disease management.

Currently, transgenic Papaya is being grown in Hawaii and accounts for more than 70% of Hawaii's Papaya acreage. SunUp and Rainbow have been widely grown in USA without any adverse effects on human health [25]. In countries such as Australia, Jamaica, Venezuela, Vietnam, Thailand, Taiwan, and Philippines, the CP gene from their geographic region has been used to develop region-specific transgenic Papaya for the control of PRSV [20]. There have been some studies upon the development of PRSV-resistant varieties of* C. papaya* through gene technology but no review article on PRSV disease management is available. Tecson Mendoza et al. [26] summarized the development of transgenic papaya technology and research activities by different countries but did not cover all areas of PRSV management. Therefore, this review paper aims to review recent development of PRSV, genomic, diversity of PRSV, molecular identification, host-range determinants and vector transmission, biosafety, major challenges, and future research directions.

## 2. The Genome of the Papaya Ringspot Virus (PRSV) 

**Figure 1:**
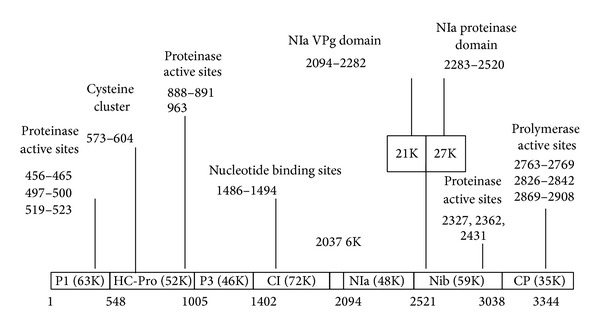
The map of the PRSV polyprotein. Specific motifs are indicated, solid bars indicate cleavage sites in the polyprotein, and the dashed line indicates the potential internal cleavage site of the NIa protein.

PRSV is of the genus* Potyvirus* and of the family Potyviridae. The genome of PRSV consists of 800–900 nm long nonenveloped flexuous filamentous particles with an ssRNA genome of about 10,324 nucleotides [27]. The virion contains 94.5% protein and 5.5% nucleic acid by weight. The PRSV genome encodes a single large protein (3,344 amino acids) which is subsequently cleaved into smaller proteins with various functions. The suggested map of the PRSV polyprotein is outlined in Figure 1.

The proposed locations of the cleavage sites predict eight to nine proteins consisting of P1 (63K), helper component (HC-Pro, 52K), P3 (46K), cylindrical inclusion protein (C1, 72K), nuclear inclusion protein a (NIa, 48K), nuclear inclusion protein b (NIb, 59K), and coat protein (CP, 35K) [27]. The P1 protein is encoded by the* Potyvirus* genome and autocatalytically cleaves. The P1 protein is the least conserved protein and can move systemically in infected plants [28]. The helper component (HC-Pro) is a multifunctional protein which mediates aphid transmission, symptom expression, long distance movement, genome amplification, and suppression of posttranscriptional gene silencing (PTGS). HC-Pro is a highly effective suppressor of RNA silencing [29]. It can affect the microRNA-mediated development pathway in plants and help in the establishment of the heterologous virus. The long distance movement and genome replication of HC-Pro depends on PTGS suppression [30]. HC-Pro is responsible for synergism between polyviruses and unrelated viruses that can cause severe symptoms and an accumulation of virus in infected leaves [31]. The C1 protein of PRSV has NTP binding, NTPase, RNA binding, and RNA helicase activity [32, 33]. The NIa has two domains defined as the N-terminal genome-linked protein (VPg) and C-terminal domain. The VPg is required for priming RNA synthesis. Nib is a codependent RNA polymerase that has been shown to have replicase activity. CP is involved in aphid transmission systemic movement and the encapsidation of the viral RNA [28]. The PRSV is divided into two major biotypes or strains based on their host range. The PRSV-W type affects cucurbits but not papaya while the PRSV-P type affects papaya and cucurbits.

## 3. The Genetic Diversity of PRSV

Knowledge of the genetic diversity of PRSV is important for effective evidence-based disease management. The genetic diversity of PRSV was observed in different regions of the world [34]. PRSV isolates and the sequence for PRSV CP genes have been presented in Table 1. Sequence diversity among isolates of the virus and their distribution are important for establishing virus origin, development, dispersion, and disease etiology, in the pursuit of effective virus disease management. There is little sequence variation among the CP genes of PRSV isolates from the USA and Australia [35, 36]. On the other hand, there is greater sequence variation amongst the CP genes of PRSV isolates from India [37] and Mexico [38]. The diversity at amino acid and nucleic acid levels was highest among the Asian population of PRSV isolates [37]. The PRSV isolates from India differed from the PRSV isolates from other countries. Bateson et al. [39] reported that the origin of PRSV was South Asia and found a greater diversity in PRSV isolates from India. The differences in PRSV were observed due to differences in the CP gene length [37]. The highest diversity of PRSV nucleotide sequences was found in the CP and HC-Pro genes collected from India [40]. PRSV might have been transported from India to America in the early 18th century and spread in 19th and 20th centuries [40]. Variation in the CP gene sequences of PRSV was observed in different parts of the world [35]. The genetic diversity of PRSV depends on geographical location. For example, the transgenic papaya incorporating CP gene (HA 5-1) isolated from USA showed resistance to PRSV infection by the severe USA isolate (HA) but did not show resistance against infection by the Australian and Thai isolates of PRSV [34].

## 4. Host Range Determinants and Vector Transmission

Plant viruses spread from cell to cell with the interaction of virus and host factors. The plant viruses enter the host cell through wound sites, mechanically or via vector mechanisms. Viruses infect the plants by two further ways: short distance (cell to cell movement) and long distance. Virus movements within the plant are dependent on host specific reactions. PRSV has limited number of hosts belonging to the families Caricaceae, Chenopodiaceae, and Cucurbitaceae. The propagation hosts of PRSV are* C. papaya, Cucurbita pepo,* and* Cucumis metuliferus*. The lesion assay hosts of PRSV are* Chenopodium quinoa* and* Chenopodium amaranticolor* [8]. There are two strains of PRSV which can be differentiated on the basis of their host range [41]. The PRSV type P (PRSV-P) can infect the papaya, whereas PRSV type W (PRSV-W) can only infect cucurbits. Both strains are closely related. Chen et al. [42] reported that the CP gene is not a determinant for the infection of papaya. The NIa and a portion of the Nib gene of PRSV were responsible for papaya infection. Mutation of P1 and HC-Pro genes resulted in the attenuation of PRSV symptoms in papaya and local lesion formation in* Chenopodium quinoa* [43] while HC-Pro is the major determinant of lesion formation in* Chenopodium quinoa.* The interaction between host and PRSV is fundamental for biological interest and for developing disease management strategies. Although, cross protection is not clearly understood at the molecular level, posttranscriptional gene silencing (PTGS) is effective for PRSV management when cultivating Papaya. The virus is transmitted by several species of aphids in a nonpersistent manner. CP and HC-Pro are required for vector transmission of PRSV. Transmission occurs when aphids feed upon infected papaya plants and subsequently feed upon healthy papaya plants.

## 5. Molecular Diagnostic of PRSV 

The most important step is identification of virus for effective PRSV control. PRSV diagnosis is very important as it exists in different strains [44]. The virus particle is very unstable and tends to aggregate with plant debris. PRSV is primarily diagnosed by assessment of symptoms; visual diagnosis is quick but it is also unreliable. The symptoms similar to PRSV can be due to the effects of micronutrient deficiency in soil and a variety of weather conditions. PRSV might be confirmed by molecular diagnosis such as ELISA, Immunocapture RT-PCR, RT-PCR, and DIBA. ELISA is widely used for rapid detection in the different parts of the world as a quick and reliable technique for PRSV detection in papaya [34, 45]. Immunocapture RT-PCR is a very reliable technique for quick determination of viruses and can detect the low concentrations of PRSV in papaya; it is a more sensitive technique than ELISA, RT-PCR, and DIBA [46]. Ruiz-Castro and Silva-Rosales [47] reported that reverse transcription and polymerase chain reaction (RT-PCR) showed reliable results for the detection of PRSV in papaya samples. Dot immune binding assay (DIBA) is useful for virus indexing, as it is a simple and cheap method for large scale virus detection [48].

## 6. Strategy for PRSV Disease Management

PRSV is the most destructive viral disease of papaya. Control of PRSV includes rouging infected plants and spraying them with aphicides. However, rouging cannot stop the spread of the disease once it is established. Similarly, spraying with aphicides is often ineffective since the virus is transmitted to the plants before the aphids are killed [49]. The PRSV disease management has been focused on developing tolerant or resistant varieties of papaya, but these varieties are rarely planted due to poor fruit quality and vigour [50]. PRSV-resistant gene is available in some wild varieties related to the* Carica* species. But the development of PRSV-resistant varieties through conventional breeding methods has been complicated due to the sexual incompatibility of wild species and cultivated papaya [51, 52]. Disease tolerance in back crosses with commercial papaya also limits this approach for PRSV disease management. Cross protection was used to control PRSV which involved the use of a mild virus strain against economic damage caused by severe strains of the same virus [6, 53]. The cross protection strategy of inoculating papaya with a mild strain of PRSV provides resistance against severe PRSV strain infection in Taiwan [54]. Cross protection depends on the availability of mild strains that can be used for effective protection against the target virus. Cross protection needs extra agricultural practice and care. However, strain specificity and the technical difficulties associated with propagating pure strains of mild forms of the virus and the unavailability of such mild strains limit the benefits of this approach [55]. Field evaluations revealed that cross protection was marginally effective for PRSV management evaluation in the field [8]. Researchers from Cornell University and the University of Hawaii initiated the development of PRSV-resistant papaya by gene technology. The concept of pathogen derived resistance was proposed by Sanford and Johnston [56] for developing resistance against pathogens. This research group has applied the concept of pathogen derived resistance which has stimulated research into obtaining virus resistance through gene technology. Pathogen derived resistance is governed either by protein-mediated or RNA-mediated methods. An alternative strategy using RNA-mediated gene silencing with transgenic plants expressing viral genes has been developed [57]. Resistance levels of PRSV differ with environmental factors and plant development stages despite of the success with this approach. Broad spectrum resistance against different PRSV isolates depends on the homology of transgenes with viral target genes and the genetic divergence of different PRSV strains which are correlated with their geographical distribution [58]. The transgenic papaya varieties resistant to PRSV against different viral strains must be developed individually for various papaya growing regions. The development of PRSV-resistant lines is generally considered the best strategy for efficient PRSV disease control in papaya for long-term protection [20].

## 7. Gene Technology for the Development of PRSV-Resistant Transgenic Papaya 

Generally crops with resistance to viral disease may be developed through genes derived from viral sequences providing pathogen derived resistance (PDR), genes from various other sources that can interfere with target virus, and natural resistance genes. The concept of pathogen derived resistance (PDR) is a new approach for PRSV management. Pathogen derived genes interfere with the replication process of viruses in their host plants in different ways. So far, PRSV-resistant transgenic papaya has been developed through coat protein (CP), RNA silencing, and replicase gene technology.

### 7.1. Coat Protein (CP) Mediated Resistance

The development of transgenic papaya to prevent infection by PRSV has been employed after the successful development of transgenic tobacco, expressing the CP gene of the tobacco mosaic virus, which showed disease resistance. Fitch et al. [59] developed transgenic papaya containing CP genes resistant to PRSV using the gene transfer system of immature zygotic embryos with a plasmid construction containing the neomycin phosphotransferase II (*npt*II) gene. This was the first result that demonstrated that CP mediated resistance can be used to control PRSV. Cheng et al. [60] developed PRSV-resistant transgenic papaya using the CP gene of the Taiwanese strain of PRSV constructed with a Ti binary vector pBGCP through* Agrobacterium *mediated transformation. Many scientists have begun to develop PRSV-resistant transgenic papaya using different explants with plasmids containing the neomycin phosphotransferase II (*npt*II) gene [42, 61–63]. The CP mediated protection of PRSV has been adopted throughout the world [64]. Researchers have preferred CP genes as the agents utilized to develop PRSV-resistant papaya [65]. However, the effectiveness of CP mediated PRSV resistance depends upon the origin of PRSV isolates. The untranslatable and translatable constructs of PRSV-CP containing genes utilized in different countries have been shown in Table 2.

Gonsalves et al. [66] used gene gun technology when transferring an untranslatable CP gene for the development of a PRSV-resistant papaya variety. This showed resistance to homologous PRSV isolates from Hawaii, Australia, Taiwan, Mexico, Jamaica, the Bahamas, and Brazil. The PRSV-resistant (Hawaiian) transgenic papaya variety SunUp was developed through transformation of somatic embryos with the CP gene of the Hawaiian PRSV strain [59]. Tennant et al. [34] developed transgenic papaya expressing the CP gene of the mild PRSV strain from Hawaii (PRV HA 5-1). This transgenic papaya showed a high level of resistance against a severe strain of PRSV (PRV-HA). Bau et al. [67] developed transgenic papaya lines expressing a CP gene with broad-spectrum resistance PRSV of different strains in different geographical areas in Taiwan. Magdalita et al. [68] developed genetically engineered papaya using the CP gene of Philippine PRSV and regenerated putative transgenic R0 plantlets, which were moderately susceptible whilst R1 plantlets were completely resistant.

### 7.2. RNA-Interference-Mediated Resistance

RNA interference (RNAi) mediated virus resistance was first discovered by Waterhouse et al. [69] against the Potato virus Y in transgenic tobacco plants. RNA mediated gene control mechanism has provided a new platform for developing molecular tools for gene functions study and crop improvements [70]. RNA silencing pathways play a role in both biotic and abiotic stress responses in plants defence against pathogens and insects that will help humankind to face the challenges of productive agriculture in the increasingly unfavourable environmental conditions associated with climate change. This technology can be used for generating disease resistance by suppressing a specific gene or genes [71]. PRSV is an RNA virus containing a single open reading frame translated into a large polyprotein that produced the final protein products [72]. RNA-mediated protection would be effective only when the transgene is highly similar to the attacking virus. The differences between geographically distinct isolates have made the creation of PRSV-resistant transgenic plants difficult. The failure of PRSV resistance has frequently involved the silencing by suppressor proteins of viral origin [73]. This problem can be overcome by the silencing suppressor protein HcPro, through an RNA-silencing mechanism within transgenic papaya. The helper component proteinase (HcPro) has been shown to be a highly effective suppressor of RNA silencing. Mangrauthia et al. [74] suggested that HcPro is an important component which needs to be taken into consideration for the development of PRSV-resistant papaya on the Indian subcontinent. The mechanism of RNA-mediated virus resistance is also referred to as homology dependency resistance to reflect the specific mechanism of posttranscriptional gene silencing (PTGS) [75, 76]. PTGS is the accumulation of 21–25 nucleotide small-interfering RNAs, the sequence-specific degradation of target mRNAs, and the subsequent methylation of target gene sequences. Tennant et al. [77] reported that mechanisms of transgenic papaya resistance against PRSV are sequence homology dependent and mediated by RNA via PTGS. They found that an untranslatable CP gene was able to confer resistance to the homologous strain of the virus isolate of PRSV by PTGS. On the other hand, the silencing suppressor was the main factor for the suppression of PRSV transgenic resistance [78]. Ruanjan et al. [73] reported that transgenic papaya showed resistant to PRSV by suppressing posttranscriptional gene silencing (PTGS).

### 7.3. Replicase Gene-Mediated Resistance

The resistance mechanism is protein-based because the resistance phenotype is influenced by mutations affecting the primary structure of the protein encoded by the transgene. Replicase genes are different in structure within various genera. Resistance created by the introduction of replicase gene was first demonstrated for the tobacco mosaic virus (TMV) in* Nicotiana tabacum* [79]. Replicase genes with mutations have been shown to be able to confer virus resistance [80]. Chen et al. [42] reported that replicase gene (RP) conferred resistance to PRSV in transgenic papaya. Wei et al. [81] reported that transgenic papaya with mutated replicase genes (RP) showed high resistance to PRSV.

## 8. Factors to Be Considered for the Adoption of Transgenic Papaya

Despite the potential and benefits of transgenic papaya, the adoption rate of transgenic papaya is extremely low. Major challenges face the adoption of transgenic papaya which include the application of the biotechnological protocols for the development of transgenic papaya, its viability as a commercial product, biosafety regulatory issues, and trade regulations. Transgenic papayas were found to have no effect on the surrounding ecology such as adjacent nontransgenic papaya trees, microbial flora, beneficial insects, or soil [82]. Hsieh and Pan [83] reported that PRSV-resistant transgenic papaya had only limited effects on the microbial life within soil. Safety assessments by researchers have suggested that transgenic papaya may not have adverse effects as regards nutritional and toxicological parameters [84]. The successful adoption of transgenic papaya depends on regulatory issues concerning biosafety and the social acceptance of the technology. PRSV-resistant transgenic papayas have been released and adopted in the USA and China [85]. Gonsalves et al. [86] reported that the adoption rate of PRSV-resistant papaya was high among famers in Hawaii. Fermin et al. [87] found a variable adoption rate of transgenic papaya in Hawaii, Jamaica, and Venezuela which was influenced by the demand for papaya, biosafety regulations, and the social acceptance of the technology. The adoption rate of transgenic papaya is still slow due to lack of engagement from farmers, who are frequently persuaded against the technology by antigenetic engineering NGO networks. Some countries are also facing opposition to transgenic papaya by Greenpeace International (NGO). Some developing countries are lacking in biosafety laws, infrastructure, and the training sufficient to carry out the regulatory testing needed prior to commercialization. There are many countries with markets that are dependent on the political and consumer demands of importing countries. Moreover, cultural awareness, societal, political, and economic factors may influence the adoption of transgenic papaya.

## 9. Environment Issues and Food Safety

Transgenic crops are subject biosafety rules due to possibly negative impacts of the plants on the environment, human, and animal health. The environmental risks may come in the form of negative effects on beneficial insects, mammals, microbes, the possibility of crossing with nontransgenic species, and their persistence in the environment. CP expressed by the transgene within a transgenic plant may enter another invading virus infecting the plant via a process of heteroencapsidation. The CP gene can carry determinants for pathogenicity and the properties of the virus in transgenic plants might be changed. So, vector nontransmissible virus in transgenic plants would be transformed into a transmissible virus through heteroencapsidation resulting in new viral epidemics. But heteroencapsidation in transgenic papaya expressing virus CP gene has limited significance in practice and environmental effects would be negligible. Recombination refers to the exchange of genetic materials between two RNA molecules during virus replication. A recombinant virus has potentially negative effects on the environment such as increasing pathogenicity, expanding host range, and changing the vector. So far, no recombination has been found in PRSV transgenic papaya in the field [23]. Research into the effects of transgenic papaya on microbiology has been conducted. Phironrit et al. [88] reported that there were no distinct differences of microbial community in soil samples collected from transgenic and nontransgenic papaya fields. On the other hand, Hsieh and Pan [83] reported that PRSV-resistant transgenic plants had a limited effect on the microbial community of the soil. Transgenic flow is a major concern amongst growers, exporters, and consumers. Fuchs and Gonsalves [23] reported that gene flow is quite low among papaya as most of the papaya was hermaphrodite in Hawaii. Manshardt [89] showed that transgenic seeds were found in 7% of nontransgenic hermaphrodite. Food safety concerns toxins and allergens. Transgenic plants have potential allergenic properties due to the protein encoded by viral sequences [23]. Yeh and Gonsalves [90] reported that there were no ill effects after the consumption of PRSV-resistant transgenic papaya. Fermín et al. [91] observed that transgene derived PRSV CP did not pose any risk of food allergies. Transgenic papaya fruit can be recognised as an equivalent substitution for traditional papaya in food safety [92]. Benzyl isothiocyanate (BITC) is considered an antinutrient which has been identified in extracts of Cruciferae, Caricaceae, and* Moringaceae* [93]. BITC may endanger the foetus in pregnant women and enhance the risk of prostate cancer [94]. The BITC value of transgenic papaya and nontransgenic papaya is similar and consequently transgenic papaya does not pose any increased threat to human health. Therefore, PRSV-resistant papaya is environmentally safe and suitable for human consumption.

## 10. Current Challenges and Future Prospects

Transgenic papaya is the most advanced technology extant for plant disease management [95]. Transgenic papaya has had a great socioeconomic impact on the Hawaiian papaya industry [86]. However, the success of transgenic papaya depends on the continued stability of transgenic resistance and the desirable horticultural characteristics of papaya. The breakdown of PRSV resistance is the main problem associated with PRSV-resistant papaya. Tennant et al. [34] reported that R_1_ transgenic papaya of line 55-1 showed narrow resistance after inoculation in greenhouse. R_1_ transgenic papaya plants were resistant to PRSV isolates from Hawaii but remained susceptible to PRSV occurring in other countries. Moreover, transgenic resistance in papaya depends on growth stage, doses of transgene, and transgene homology [34]. Resistance to PRSV is positively correlated with a degree of homology between the CP of infecting virus and transgene [8]. For example, 97–100% of the sequence homology to transgene CP was found in PRSV isolates from Hawaii whilst 89–93% sequence homology of transgene CP was found in PRSV isolates from elsewhere [8]. Tripathi et al. [8] reported that the CP expression of transgenic papaya plants was lower in homozygous SunUp than hemizygous Rainbow. Transgenic resistance in Rainbow and SunUp has proven stable for nearly 10 years in Hawaii but resistance may breakdown in regions where new virus strains exist. There is great genetic diversity within PRSV isolates from different regions of the world. PRSV-resistant transgenic papaya faces major difficulties as no current strain has resistance against geographically distinct isolates. It is important that researchers monitor the PRSV population and its diversity to ensure the successful disease management of papaya. On the other hand, posttranscriptional gene silencing (PTGS) technology is possibly a more powerful and effective method for the development of PRSV-resistant transgenic papaya. Therefore, country-specific varieties of PRSV-resistant transgenic papaya should be developed through PTGS technology using geographically distinct PRSV isolates.

## 11. Conclusion 

PRSV is the major threat for papaya production. Transgenic papaya via gene technology has been used for PRSV disease management. In this review, we find that PRSV-resistant papaya varieties have been developed using CP genes or RNA interference. The genetic diversity of PRSV has been identified throughout the world. The breakdown of PRSV resistance is the major challenge facing transgenic papaya cultivation. Although, the gene flow of PRSV-transgenic papaya is low, research towards minimizing this problem should be conducted. The adoption of PRSV-resistant transgenic papaya is still slow and it depends upon the demand for papaya, biosafety regulations, and social acceptance of the technology. Recent studies indicate that PRSV-resistant transgenic papaya is environmentally safe and has no adverse effects on human health. Posttranscriptional gene silencing (PTGS) technology may be suitable for the development of PRSV-resistant transgenic papaya in future. This review suggests that papaya producing countries should develop PRSV-resistant transgenic papaya using their own PRSV isolates through posttranscriptional gene silencing technology.

## Figures and Tables

**Table 1:** PRSV isolates and sequence for PRSV CP genes.

Name	Biotype	Origin	Gene Bank accession no.	Reference
USP-HW	P	USA	X67673	[39]
USW-FL	W	USA	D00594	[36]
USP-FL	P	USA	AF196839	[64]
AU-P	P	Australia	U14738	[35]
BD	P	Australia	U14736	[35]
BUN	P	Australia	U14737	[35]
WP	P	Australia	U14740	[96]
TWP-2	P	Taiwan	AB044341	[39]
TWP-YK	P	Taiwan	X78557	[44]
THP-11	P	Thailand	U14743	[39]
THW-03	W	Thailand	AF506895	[39]
THW-04	W	Thailand	AF506894	[39]
THP-14	P	Thailand	AF506898	[39]
THP-13	P	Thailand	AF506899	[39]
THP-12	P	Thailand	AF506900	[39]
INP-BR	P	India	AF305545	[38]
INW	P	India	AF063221	[39]
INP	P	India	AF063220	[39]
VNP-02-29	P	Vietnam	AF506862-89	[39]
VNW-30-32	W	Vietnam	AF506846-48	[39]
SRP	P	Sri Lanka	U14741	[35]
BZP-2	P	Brazil	AF344640	[39]
BZP-9	P	Brazil	AF344647	[39]
CHP	P	China	AF243496	[96]
JAP	P	Japan	AB044339	[39]
PHP-01	P	Philippines	AF506902	[39]
ChT-11	P	Mexico	AJ012650	[38]
VTB-6	P	Mexico	AJ012649	[38]

**Table 2:** Transgenic papaya developed by various research groups through gene technology.

Country	Cultivar	Construct	Type of coat protein	Transformation	Transgene expression	References
Australia	Local variety	uidA leader + CaMV35S promoter + PRSV Bridgeman Downs cp gene from Q/S start with stop codon in the middle of sequence	Translatable cp	Biolistics	*cp* not detected in ELISA and low levels of *cp * detection in northern analysis	[61]
Brazil	Sunrise solo, Sunset solo	CaMV35S + CMV leader + PRSV Bahia cp gene from Q/S start	Translatable cp	Biolistics	Low to high levels *cp* detected in ELISA	[97]
Brazil	Sunrise solo, Sunset solo	CaMV35S + CMV leader + PRSV Bahia cp gene from Q/S start	Untranslatable cp	Biolistics	Not tested	[97]
Florida	CV.F65	*uidA* leader + CaMV35S promoter + PRSV HIK cp gene from Q/S start	Translatable cp	*Agrobacterium *	*cp* is not detected in northern analysis	[98]
Florida	CV.F65	*uidA* leader + CaMV35S promoter + PRSV HIK cp gene from Q/S start in antisense	Untranslatable cp	*Agrobacterium *	*cp* is not detected in northern analysis	[98]
Florida	CV.F65	*uidA* leader + CaMV35S promoter + PRSV HIK cp gene from Q/S start with frame shift mutation	Untranslatable cp	*Agrobacterium *	*cp* is not detected in northern analysis	[98]
Florida	CV.F65	*uidA* leader + CaMV35S promoter + PRSV HIK cp gene from Q/S start with 3 in frame stop	Untranslatable cp	*Agrobacterium *	*cp *is not detection in northern analysis	[98]
Hawaii	Sunset solo	CMV leader + 16 aa CMV cp + PRSV HA 5-1 cp gene from Q/S start	Translatable cp	Biolistics	Low to high levels *cp *detected in ELISA and cp RNA detected by northern analysis	[99, 100]
Hawaii	Sunset solo	CaMV35S + CMV leader + PRSV Caymanas untranslatable cp	Untranslatable cp	Biolistics	*cp* RNA detected in northern analysis	[101]
Jamaica	Sunrise solo	CaMV35S + CMV leader + PRSV Caymanas untranslatable cp	Untranslatable cp	Biolistics	*cp* RNA detected in northern analysis	[62, 99]
Jamaica	Sunrise solo	CaMV35S + CMV leader + PRSV Caymanas cp from Q/S start	Translatable cp	Biolistics	*cp* RNA detected in northern analysis	[62, 99]
Taiwan	Tainung no. 2	uidA leader + CaMV35S promoter + PRSV YK cp gene from Q/S start	Translatable cp	Biolistics	*cp* transcript detected in northern analysis	[58, 78]
Thailand	KhakDum	CaMV35S + uidA leader + PRSV Ratchaburi province cp	Translatable cp	Biolistics	*cp* detected in western analysis	[73, 102]
Venezuela	Thailand red	CaMV35S + CMV leader + PRSV EV & VE from Q/S start	Translatable cp	*Agrobacterium *	*cp* RNA is not detected ELISA and low level cp detected in northern analysis	[87]
